# Unravelling the Impact of Diabetes on Insulin-like Growth Factor (IGF) Axis Signalling in Mesenchymal Stem Cells Isolated from the Bone of Knee Joints

**DOI:** 10.3390/bioengineering13091043

**Published:** 2026-09-08

**Authors:** Nancy Hussein, Josephine L. Meade, Hemant Pandit, Elena Jones, Reem El-Gendy

**Affiliations:** 1 Division of Oral Biology, School of Dentistry, University of Leeds, Leeds LS9 7TF, UK; nancy_sobeih@mans.edu.eg (N.H.); j.l.meade@leeds.ac.uk (J.L.M.); 2 Department of Oral Medicine and Periodontology, Faculty of Dentistry, Mansoura University, Mansoura 3551, Egypt; 3 Leeds Institute of Rheumatic and Musculoskeletal Medicine, School of Medicine, University of Leeds, Leeds LS9 7TF, UK; h.pandit@leeds.ac.uk (H.P.); e.jones@leeds.ac.uk (E.J.); 4 Department of Oral Pathology, Faculty of Dentistry, Suez Canal University, Ismailia 41522, Egypt

**Keywords:** type 2 diabetes mellitus, bone marrow mesenchymal stem cells, IGF axis, bone regeneration

## Abstract

Diabetes mellitus (T2DM) represents a major health problem with several potentially life-threatening complications including cardiovascular disease and osteopathy. Bone marrow mesenchymal stem cells (BM-MSCs) are a promising candidate for bone regeneration, and the insulin-like growth factor (IGF) axis plays a fundamental role in both bone regeneration and stem cell biology. Still, its expression profile is yet to be assessed in diabetic BM-MSCs. This study investigated IGF axis gene and protein expression in BM-MSCs isolated from the knee joints of diabetic and non-diabetic donors. BM-MSCs were cultured under basal and osteogenic conditions for 1, 2 and 3 weeks. Relative expression levels of the IGF axis genes were assessed using qPCR while protein concentrations of IGFBP-2, -3 and -4 were assessed using ELISA. Diabetic BM-MSCs showed lower mRNA levels of IGF binding proteins (IGFBP)-2, -3 and -4 but both cell populations displayed comparable protein levels. Moreover, the osteogenic cultures of diabetic and non-diabetic BM-MSCs equally demonstrated a trend of IGFBP-2 upregulation along with IGF-1 and IGFBP-5 downregulation. Non-diabetic BM-MSCs showed significant time-dependent increases in IGFBP-3 and -4 concentrations in basal and osteogenic cultures respectively. These molecules could be addressed to improve the regenerative potentials of BM-MSCs particularly under diabetic conditions, and further research is warranted into the possible roles of IGFBPs proteinases and their inhibitors in bone biology.

## 1. Introduction

Type 2 diabetes mellitus (T2DM) is characterised by chronic hyperglycaemia and hyperinsulinemia caused by insulin resistance. This disruption in insulin signalling and glucose metabolism leads to abnormal lipid and protein metabolism as well [1]. The global diabetes prevalence in adults (20–79-year-olds) was estimated to be 10.5% (around 537 million people) in 2021, which would increase to 12.2% (around 783 million) in 2045, with T2DM representing the majority of these numbers [2]. Diabetes is a major risk factor of both fragility fractures and post-fracture complications such as delayed healing and infection [3], as well as periodontitis [4]. Bone marrow mesenchymal stem cells (BM-MSCs) are multipotent cells with immunomodulatory and proangiogenic potentials as well and have been widely used in regenerative medicine including liver, cartilage, bone and cardiac regeneration [5]. Systemic administration of autologous BM-MSCs in patients with diabetes showed no adverse effects [5]. However, impaired functions of BM-MSCs isolated from patients with diabetes have been reported mainly due to the disrupted architecture of the BM-MSCs niche in the bone marrow owing to increased fat deposition and microangiopathy [6]. Different strategies have been proposed to correct the diabetes-induced defects of BM-MSCs, including antioxidants, anti-inflammatory drugs and growth factors [7].

The insulin-like growth factor (IGF) axis is composed of two ligands (IGF-1 and -2), two receptors (IGF-1 R and IGF2-R) and six binding proteins (IGFBP-1 to -6) [8]. IGF-1 and -2 represent the most abundant growth factors in the skeleton and play essential roles in bone growth and maintenance through binding to IGF1-R and subsequent regulation of both osteoblast and osteoclast differentiation and function [9].

Emerging evidence suggests a link between IGF axis proteins and OA in T2DM patients. Higher serum IGF-1 levels were causally associated with higher risk of knee OA [10]. IGFBP-3 plays a central role in the regulation of multiple signalling pathways and intercellular communication within the knee joint, and this role gets more nuanced in diabetic OA since IGFBP3 activity is controlled by endocrinal factors [11].

Expression of the IGF axis genes has been assessed in dental pulp stem cells (DPSCs) [12], and IGFBP-2 and -3 were shown to control osteogenic differentiation of DPSCs [13]. This study aimed at investigating the expression of IGF axis genes in non-diabetic (ND) and diabetic (D) BM-MSCs cultured under basal and osteogenic conditions after 1, 2 and 3 weeks of culture.

## 2. Materials and Methods

### 2.1. BM-MSCs Isolation and Culture

Human bone samples from ND and D patients (*n* = 3 for each) undergoing joint replacement surgeries due to primary osteoarthritis (OA) of the knee joints at Chapel Allerton Hospital were used for isolation of human BM-MSCs. The age of ND donors ranged from 64 to 86 yrs old while the D donors age ranged from 81 to 85 yrs old. Two of the D donors had HbA1c of 45 and 47 mmol/mol. The samples were collected under approval from the Yorkshire and Humberside National Research Ethics Committee (Reference number 14/YH/0087) and with the patients’ informed written consent.

Cells were isolated, expanded and characterised using multiparameter flow cytometric analysis as described earlier [14]. Briefly, BM-MSCs were isolated from the knee joints using enzymatic digestion and the cell suspension was then centrifuged to get the cell pellet which was resuspended in basal culture media. The cells were kept in an incubator at 37 °C and 5% CO_2_. After 2 days, the culture media was fully discarded, and the adherent cells were washed with PBS (Lonza BioWhittaker, Slough, UK) twice. For passaging, cultured cells with 80% confluence were washed with PBS twice and detached using trypsin-EDTA 0.25% (*w*/*v*) solution (Sigma-Aldrich, Gillingham, UK) for 10 min.

### 2.2. Osteogenic Differentiation of BM-MSCs

ND and D BM-MSCs at passage 2–4 were cultured under basal conditions: complete media—α Modified Eagle media (αMEM, Lonza BioWhittaker, Slough, UK) supplemented with 20% foetal bovine serum, 1% Penicillin/Streptomycin and 1% L-glutamine (all from Sigma-Aldrich, Gillingham, UK); and osteogenic conditions: complete media supplied with 10nM dexamethasone, 50 µg/mL L-ascorbic acid and 5mM β-glycerophosphate (all from Sigma-Aldrich, Gillingham, UK) as described elsewhere [15]. Briefly, the BM-MSCs were seeded into T25 flasks (Corning^®^, Ewloe, UK) (n = 1 × 3 diabetic and 3 non-diabetic donors), at a density of 1 × 10^5^ cells per flask. After 1, 2 and 3 weeks of culture, the culture media was collected and stored at −80 °C pending the ELISAs. The cells were then washed and lysed for RNA extraction and subsequent cDNA synthesis. For baseline (T0) controls, a total of 1 × 10^5^ trypsinised cells were resuspended in lysis buffer at the experiment setup and immediately frozen at −80 °C.

### 2.3. Gene Expression Assessment Using qPCR

Total mRNA was extracted using RNeasy^®^ Mini Kit with on-column gDNA digestion using an RNase-Free DNase Set (both Qiagen, Manchester, UK) according to the manufacturer’s protocol. mRNA quality was evaluated by measuring the A260/280 ratios and then the mRNA was reverse-transcribed into cDNA using the High-capacity RNA-to-cDNA™ kit (Applied Biosystems; ThermoFisher Scientific, Loughborough, UK) according to the manufacturer’s protocol. qPCR reactions were run in duplicates using a 20 µL mixture of TaqMan^®^ Master Mix (Applied Biosystems; ThermoFisher Scientific, Loughborough, UK), TaqMan^®^ probes (Applied Biosystems; ThermoFisher Scientific, Loughborough, UK—Supplementary Table S1), cDNA and UltraPure™ DNase/RNase-Free distilled water (ThermoFisher Scientific, Loughborough, UK) on Roche 480 Light Cycler as described earlier [16]. At each time point, the ∆Ct method with Hypoxanthine -Guanine Phosphoribosyltransferase 1 (*HPRT-1*) serving as the housekeeping gene (HKG) was used for assessing the relative expression of the IGF axis genes as described earlier [14]. Genes of interest were normalised to *HPRT-1* using the following equations:

∆Ct = (gene of interest − housekeeping gene)

The relative change in gene expression = 2^−∆Ct^

### 2.4. Assessing IGFBPs Levels in Conditioned Media Using ELISA

The concentrations of IGFBP-2, -3 and -4 were assessed in conditioned media of 1-, 2- and 3-week basal and osteogenic cultures of ND and D BM-MSCs. These concentrations were measured using human IGFBP-2 and IGFBP-3 Quantikine^®^ ELISA kits (R&D systems, Abingdon, UK) and an IGFBP-4 DuoSet^®^ ELISA kit (R&D systems, Abingdon, UK) respectively according to the manufacturer’s protocol as described earlier [13]. At the designated time points, conditioned media was collected, centrifuged at 148× *g* at room temperature for 5 min and then aliquoted to prelabelled 1.5 mL Eppendorf tubes. These aliquots were stored at −80 °C pending the ELISA analysis.

### 2.5. Statistical Analysis

Data were analysed using GraphPad Prism software (v 9.2.0, Dotmatics, Boston, MA, USA) and are presented as mean ± SEM. Matched data (basal and osteogenic cultures of BM-MSCs from donors with the same diabetic status) were compared using paired *t* test and non-matched data were compared using unpaired *t* test. Repeated measures ANOVA (RM-ANOVA) was used to compare different time points. Furthermore, 3-way ANOVA was used for the overarching analysis of all variables across the 4 experimental setups. For all comparisons, *p* values < 0.05 were considered significant.

## 3. Results

### 3.1. Gene Expression of IGF Axis in Diabetic and Non-Diabetic BM-MSCs

**Figure 1:**
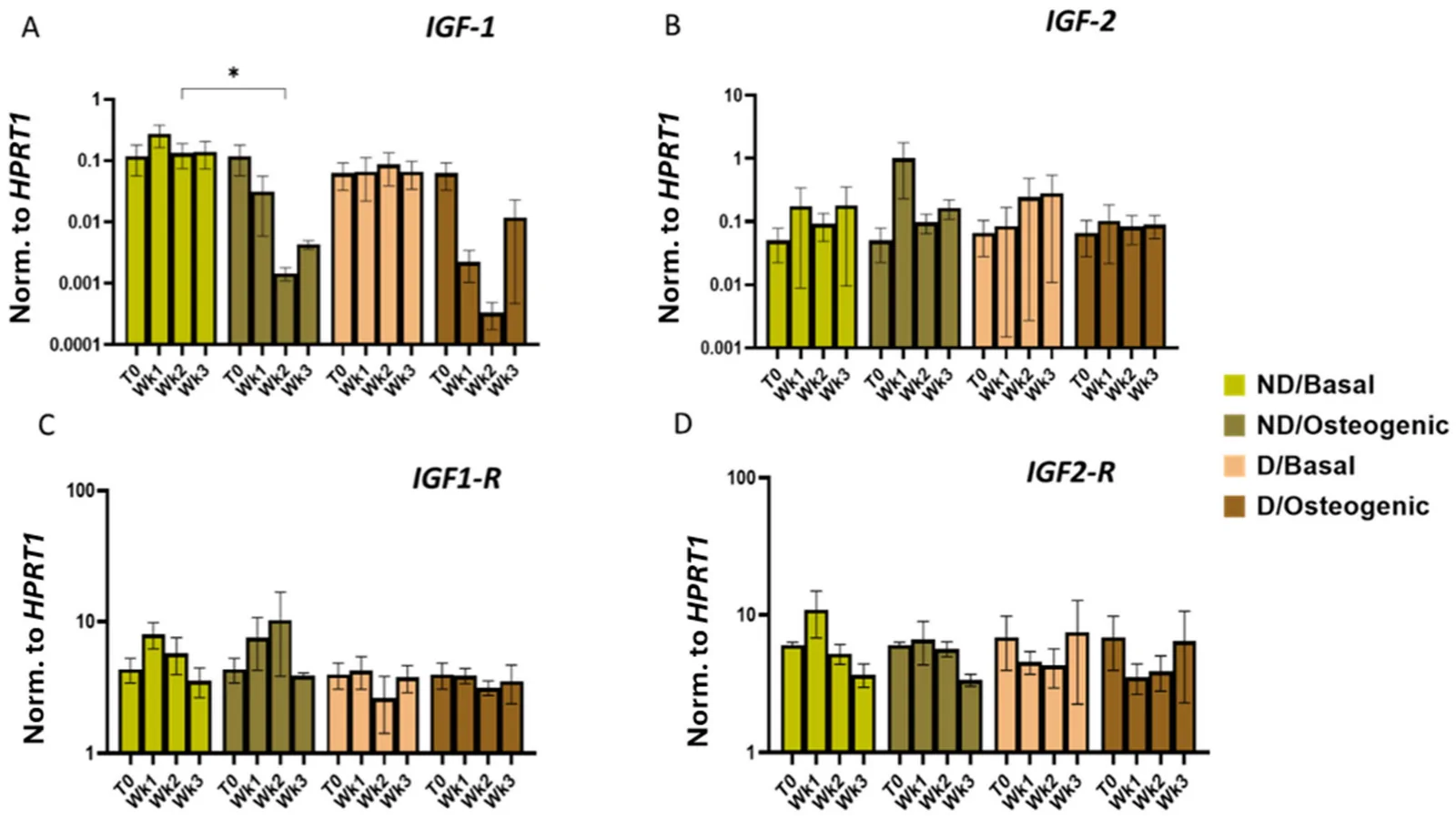
Relative changes in gene expression of (**A**) *IGF-1*, (**B**) *IGF-2*, (**C**) *IGF1-R* and (**D**) *IGF2-R* in ND and D BM-MSCs at T0, basal and osteogenic conditions at Wk1, Wk2 and Wk3 time points. Data presented as mean ± SEM (n = 3) and was analysed using unpaired *t* test for unmatched groups, paired *t* test for matched groups and repeated measures ANOVA for comparing time points. * *p* < 0.05.

*IGF-1*, *IGF-2* and their receptors showed no significant differences or trends of differences between ND and D BM-MSCs. However, ND and D cell populations displayed a trend of *IGF-1* downregulation in osteogenic vs. basal cultures across weeks 1–3. Among these differences, only the one noted in osteogenic vs. basal cultures of ND BM-MSCs after 2 weeks was statistically significant (*p* < 0.05) (Figure 1A,B).

**Figure 2:**
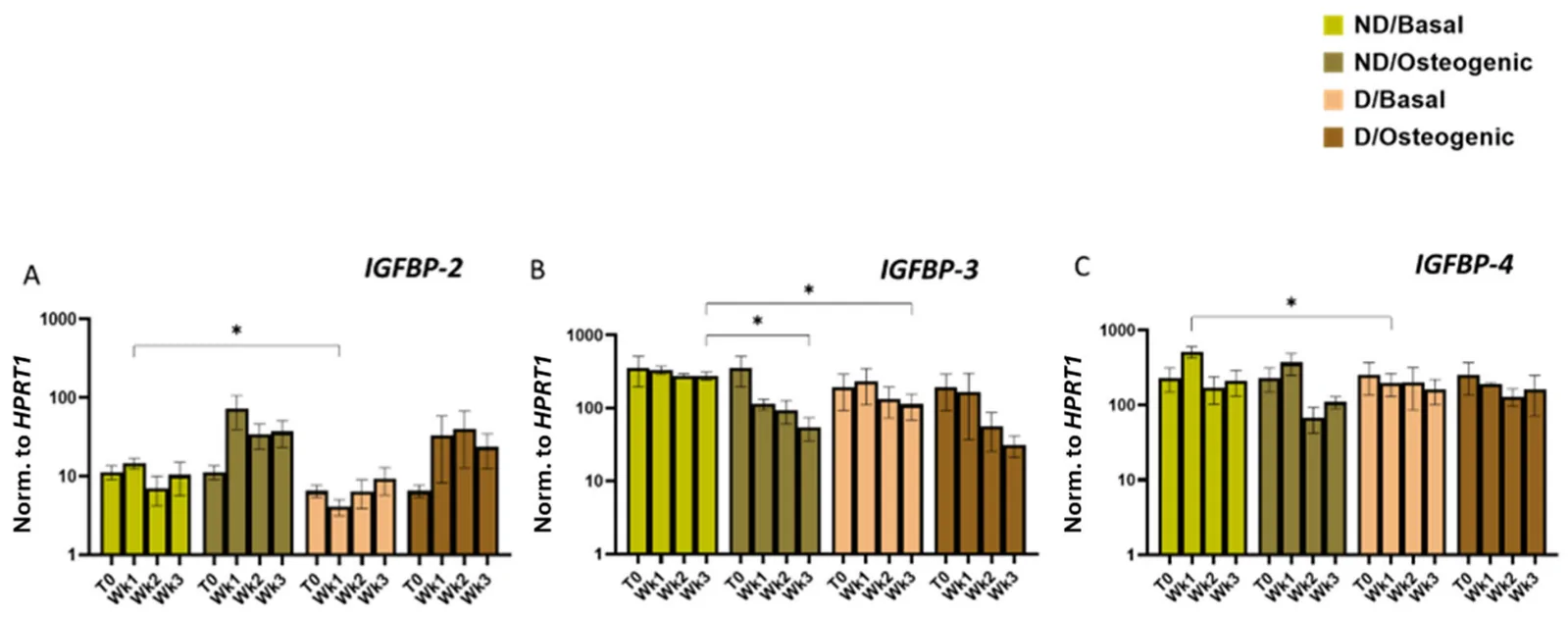
Relative changes in gene expression of (**A**) *IGFBP-2*, (**B**) *IGFBP-3* and (**C**) *IGFBP-4* in ND and D BM-MSCs at T0, basal and osteogenic conditions at Wk1, Wk2 and Wk3 time points. Data presented as mean ± SEM (n = 3) and was analysed using unpaired *t* test for unmatched groups, paired *t* test for matched groups and repeated measures ANOVA for comparing time points. * *p* < 0.05.

**Figure 3:**
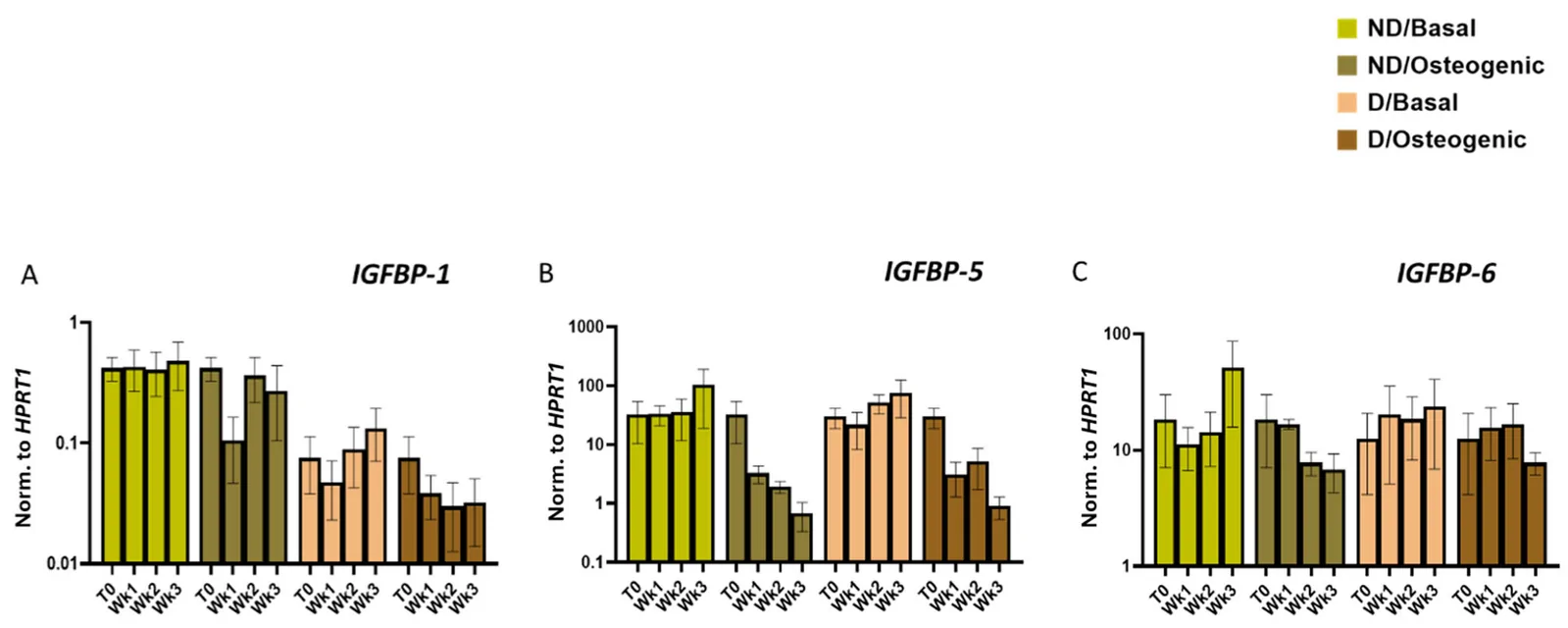
Relative changes in gene expression of (**A**) *IGFBP-1*, (**B**) *IGFBP-5* and (**C**) *IGFBP-6* in ND and D BM-MSCs at T0, basal and osteogenic conditions at Wk1, Wk2 and Wk3 time points. Data presented as mean ± SEM (n = 3) and was analysed using unpaired *t* test for unmatched groups, paired *t* test for matched groups and repeated measures ANOVA for comparing time points.

The D BM-MSCs showed a statistically significant lower expression of *IGFBP-2* at Wk1 basal cultures (Figure 2A), *IGFBP-3* at Wk3 basal cultures and *IGFBP-4* at Wk1 basal cultures compared to the ND cells (*p* < 0.05) (Figure 2B,C). *IGFBP-2* displayed a trend of upregulation in osteogenic vs. basal cultures of D (at Wk1 and Wk3) as well as ND (at Wk2 and Wk3) cells (Figure 2A). *IGFBP-3* was markedly downregulated in osteogenic vs. basal cultures of ND BM-MSCs after 3 weeks of culture (*p* < 0.05) (Figure 2B). *IGFBP-5* exhibited a trend of downregulation in the osteogenic cultures of both cell populations across weeks 1–3 relative to their basal counterparts (Figure 3B). No genes showed time-dependent changes in their expression profiles.

Analysis using three-way ANOVA has shown that the diabetic status of the BM-MSC donors had a statistically significant impact on the expression of *IGF-1*, *IGF1-R*, *IGFBP-1* and *-3*. The culture media influenced the expression of *IGF-1*, *IGFBP-2*, *-3* and *-5*, while culture duration significantly impacted the expression of *IGFBP-4* (Table 1).

### 3.2. Quantification of IGFBPs in Culture Supernatant of Non-Diabetic and Diabetic BM-MSCs

**Figure 4:**
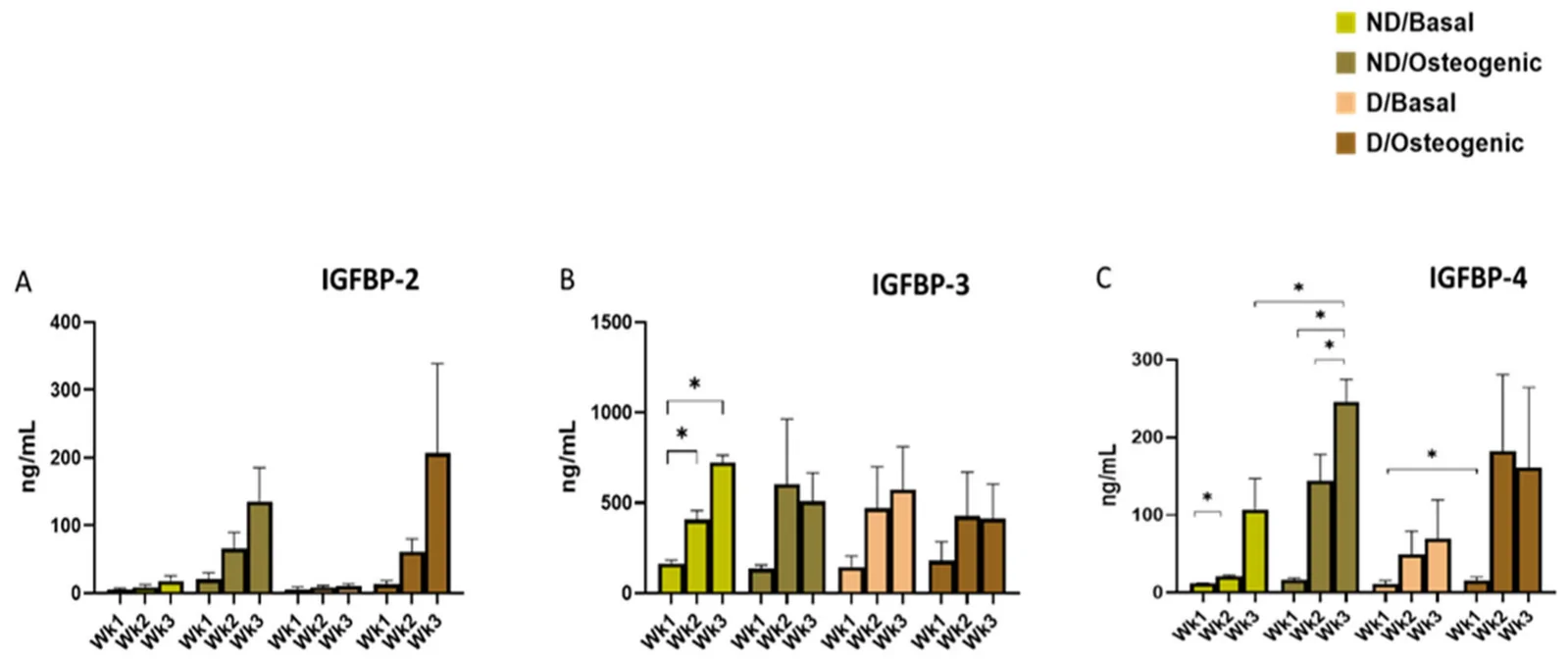
Protein levels of (**A**) IGFBP-2, (**B**) IGFBP-3 and (**C**) IGFBP-4 in conditioned media of ND and D BM-MSCs cultured under basal and osteogenic conditions at Wk1, Wk2 and Wk3 time points. Data presented as mean ± SEM (n = 3) and was analysed using unpaired *t* test for unmatched groups, paired *t* test for matched groups and repeated measures ANOVA for comparing time points. * *p* < 0.05.

Given that IGFBP-2, -3 and -4 displayed statistically significant reduced expression levels in D vs. ND BM-MSCs at specific time points, the protein levels of these molecules were examined in the culture supernatant of both cell populations. However, these IGFBPs showed no statistically significant differences between ND and D cell populations at any time point/culture condition. Nevertheless, IGFBP-2 had a trend of higher levels in osteogenic vs. basal cultures of both ND and D BM-MSCs (Figure 4A). Basal cultures of ND cells demonstrated markedly elevated concentrations of IGFBP-3 at Wk3 vs. Wk1 and Wk3 vs. Wk2 (*p* < 0.05) (Figure 4B). ND cells showed significantly greater concentrations of IGFBP-4 at Wk2 vs. Wk1 basal cultures and at Wk3 vs. Wk1 and Wk3 vs. Wk2 osteogenic cultures (*p* < 0.05) (Figure 4C). Likewise, D BM-MSCs demonstrated significantly greater IGFBP-4 quantities in osteogenic culture supernatant relative to the basal controls after 1 week of culture (*p* < 0.05). ND BM-MSCs cultured for 3 weeks demonstrated markedly elevated IGFBP-4 concentrations in osteogenic culture supernatant relative to their basal counterparts (*p* < 0.05) (Figure 4C).

## 4. Discussion

To the best of our knowledge, the present study was the first attempt to evaluate IGF axis expression in BM-MSCs isolated from the knee joints of T2DM patients. In this study, the gene and protein expression of IGF axis members was investigated in DBM-MSCs and NDBM-MSCs cultured under both basal and osteogenic conditions, at 1, 2 and 3 weeks. These time points were selected to capture changes in IGF axis expression across the early, intermediate and late stages of osteogenic differentiation [17].

The BM-MSCs investigated in this study were isolated from already osteoarthritic knee joints of patients undergoing knee replacement surgery. A major advantage of this method would be making use of cells and tissues that otherwise would go to medical waste. However, this might have added another layer of complexity to the analysis that may be considered a limitation to this study. An alternative approach could be isolating BM-MSCs from non-osteoarthritic joints of diabetic and non-diabetic patients undergoing surgeries for fracture fixation or other traumatic incidents, yet the availability of such samples is quite challenging and unpredictable.

Aligned with earlier experimental evidence reporting that *IGF-1* and *-2* were both expressed by human osteoblasts [18], untreated ND and D BM-MSCs in the present study expressed *IGF-1* and *-2* at baseline. Overall, no notable differences in *IGF-1* levels in ND vs. D BM-MSCs were observed, whether at T0, in basal or osteogenic cultures. On the contrary, previous work demonstrated *IGF-1* downregulation in human DPSCs cultured in high glucose (HG) basal and osteogenic media [19]. Nevertheless, using media supplemented with HG does not fully replicate the T2DM microenvironment which also entails hyperinsulinemia, hyperlipidaemia, inflammatory cytokines and advanced glycated end products (AGEs) [20,21].

Our data showed that *IGF-1* demonstrated a trend of downregulation in the osteogenic cultures of both ND and D BM-MSCs across weeks 1–3 relative to the basal counterparts, with significant difference observed with ND BM-MSCs cultured for 2 weeks (*p* < 0.05). This pattern is plausibly linked to dexamethasone supplementation in the osteogenic media and is consistent with the downregulation of *IGF-1* observed in rat osteoblasts cultured with cortisol [22]. Nonetheless, MC3T3 osteoblasts demonstrated a progressive (albeit not significant) increase in *IGF-1* expression from day 5 to day 21 of culture [23]. Rat osteoblasts in a different study have also shown *IGF-1* upregulation at days 3 and 6 [24], and in both investigations the osteogenic media supplementation relied only on glycerophosphate and ascorbic acid.

IGF-1, IGF-2 and insulin can all bind IGF-1R, with IGF-1 exhibiting the strongest affinity, and the GH/IGF-1/IGF1-R axis is essential for normal growth and development [25]. This could probably explain the fairly stable expression profile of *IGF1-R* across ND and D BM-MSCs under basal and osteogenic conditions. Supporting this, murine MSCs deficient in IGF-1R failed to undergo osteoblastic differentiation, while mice with knocked out *IGF1-R* exhibited reduced bone mass relative to their wild-type controls [26]. While we observed that *IGF1-R* demonstrated similar expression patterns among ND and D BM-MSCs, *IGF1-R* was downregulated on both transcript and protein levels in HG basal and osteogenic cultures of DPSCs [19].

The expression levels of *IGF-1* and *-2* remained consistently low in both ND and D BM-MSCs along with relatively higher expression levels of their receptors. This observation partially aligns with the findings of Al-Khafaji et al. [12], where *IGF-2*, but not *IGF-1*, was identified in DPSC cultures. Al-Khafaji et al. [12] proposed that IGFs could instead originate from the dense vascular supply of the dental pulp or are alternatively secreted by adjacent cells, acting on DPSCs via paracrine signalling. These mechanisms could equally be applicable to BM-MSCs. Furthermore, the osteoarthritic status of BM-MSCs used in the present study and/or the donors’ relatively advanced age could have promoted these diminished expression levels of both IGFs.

After 1 week of culture, *IGFBP-2* expression levels were markedly reduced in basal cultures of D BM-MSCs relative to their ND counterparts (*p* < 0.05). Considering the established proangiogenic capacities of IGFBP-2 [27], this pattern of *IGFBP-2* diminished expression in D BM-MSCs could underline the impaired angiogenic potentials and the ensuing poor wound healing commonly reported in tissues of patients with T2DM [28]. The angiogenic potentials of adipose tissue mesenchymal stem cells (AT-MSCs) from patients with T2DM and cardiovascular disease (CVD) [29], or even T2DM alone [30], were reduced. Similar changes were noted when BM-MSCs were treated with serum from T2DM patients [31,32] or HG [33]. Our previous study demonstrated a trend towards reduced expression of osteogenic marker genes (*ALP*, *POSTN*, *RUNX2*, and *OCN*) in T2DM-derived BM-MSCs, although these differences did not reach statistical significance. The present findings of reduced *IGFBP-2* gene expression in D-BM-MSCs are consistent with the reported role of IGFBP-2 in promoting osteogenic differentiation, suggesting that T2DM may impair IGFBP-2 signalling and thereby contribute to the reduced osteogenic potential of these cells [14].

Our results also showed a trend of *IGFBP-2* upregulation in osteogenic vs. basal cultures of both D (at time points Wk1 and Wk3) and ND (Wk2 and Wk3) cells. These observations are aligned with prior research demonstrating *IGFBP-2* upregulation during osteogenic differentiation of MSCs. For instance, BM-MSCs treated with 10–7 M (100 nM) dexamethasone exhibited upregulation of both *IGF-2* and *IGFBP-2* relative to their basal counterparts [34]. Similarly, Alkharobi et al. [13] reported that *IGFBP-2* was upregulated during osteogenic differentiation of DPSCs from both healthy and carious teeth. This *IGFBP-2* upregulation was accompanied by *IGFBP-3* downregulation, and these two changes promoted the pro-osteogenic effect of IGF-1 added to DPSC cultures [13]. Cheng et al. [35] noted that *IGFBP-2* was upregulated in human BM-MSC cultures supplemented with dexamethasone at days 5 and 7; and Jia et al. [36] also reported that dexamethasone treatment caused *IGFBP-2* upregulation in murine osteoprogenitor-containing bone cell populations at days 8, 14 and 20, approximately corresponding to the time points examined in the present study.

*IGFBP-3* showed significantly lower expression levels by D vs. ND cells after 3 weeks of basal culture (*p* < 0.05). These findings are in line with previous reports lower *IGFBP-3* expression in subcutaneous adipose tissue of diabetic patients, a phenomenon thought to reflect the impaired adipocytes’ differentiation capacities, ensuing fat accumulation and insulin resistance [37]. Osteogenic cultures of ND BM-MSCs demonstrated lower expression levels of *IGFBP-3* relative to their basal controls after 3 weeks (*p* < 0.05), with comparable trends after 1 and 2 weeks of culture. Similar to IGF-1, this could be attributable to adding dexamethasone to the osteogenic media. Prior studies reported diminished *IGFBP-3* expression in human BM-MSCs [35] and rat hepatocytes [38] treated with dexamethasone. IGFBP-3 is the predominant IGFBP in serum, sequestering the majority of the IGFs and decreasing their availability to bind their receptors [39]. Therefore, *IGFBP-3* diminished transcript levels in osteogenic media could at least in theory increase the pool of free IGFs available to bind their receptors. Ultimately, this would enhance the pro-proliferation and pro-mineralisation potentials of IGF-1 and -2 during osteogenic differentiation of ND BM-MSCs.

After 1 week of culture, *IGFBP-4* expression levels were markedly lower in the basal cultures of the D BM-MSCs relative to their ND counterparts (*p* < 0.05). To our knowledge, no prior studies have examined *IGFBP 4* expression in BM MSCs derived from T2DM patients. Nonetheless, vascular smooth muscle cells of porcine origin cultured under HG also demonstrated similar transcript levels but lower protein quantification of IGFBP-4 relative to their controls. These changes were associated with enhanced IGFBP-4 proteolysis and greater IGF-1 concentrations in HG cultures, potentially driving endothelial proliferation and contributing to the macroangiopathic complications and CVD in patients with T2DM [40].

Across weeks 1–3, *IGFBP-5* tended to be downregulated in the osteogenic cultures of ND and D BM-MSCs relative to their basal controls. This contrasts with prior research reporting *IGFBP-5* upregulation in BM-MSCS, AT-MSCs and periodontal ligament stem cells (PDLSCs) [41] cultured using a commercial osteogenic differentiation kit possibly differing in supplement composition from the current study. The downregulation noted herein may be linked to dexamethasone as described above with other IGF axis genes. Dexamethasone-treated cultures of human BM preosteoblastic cells exhibited *IGFBP-5* downregulation and higher concentrations of IGF-2 and these two changes collectively enhanced the gross pro-osteogenic capacities of dexamethasone [35].

Nevertheless, exogenous IGFBP-5 enhanced the osteogenic and odontogenic differentiation capacities of multiple types of MSCs, including BM-MSCs and PDLSCs [42], as well as DPSCs [43]. These paradoxical impacts of exogenous vs. endogenous IGFBP-5 have been described elsewhere, where the former preferentially binds IGF-1 and modulates the IGF-1/IGF1-R axis, while the latter may interact with potential nuclear receptors [44]. Indeed, IGFBP-5 could associate with the nuclear vitamin D receptor in human osteosarcoma cells lines [45]. Still, *IGFBP-5* overexpression suppressed expression of osteogenic markers in MC3T3 cells [46]; and exogenous addition and adenoviral transfection of IGFBP-5 equally inhibited the pro-osteogenic effects of BMP-2 in mice embryonic fibroblasts [47]. Whether IGFBP-5 treatment would inhibit osteogenic differentiation and/or expression of osteogenic markers in human BM-MSCs remains to be fully investigated.

Despite significant reductions in the transcript levels of *IGFBP-2*, *-3*, and *-4* in D BM MSCs compared with ND cells at particular time points, their protein concentrations did not differ between ND and D cell culture supernatant across all culture conditions. This indicates that lower transcript levels did not necessarily translate into diminished protein concentrations and that D BM-MSCs retained similar abundance of these proteins relative to their ND counterparts. The assumption that mRNA levels dictate protein outputs may be oversimplified [48]. In reality, protein expression profiles are shaped by multiple regulatory mechanisms including post-transcriptional, translational and protein degradation processes [49]. This divergence between transcriptomic and proteomic data has been widely documented [50]. Since IGFBPs bind IGFs as well as cell receptors and extracellular matrix proteins to mediate the IGFs’ independent functions [51], some IGFBPs were possibly bound to cell surface receptors and thus were not measured by the ELISAs which detect IGFBPs in cell culture supernatant. Furthermore, the assays possibly quantified free rather than total IGFBPs, excluding complexed molecules.

The current study also demonstrated a trend of progressive, time-linked rise in IGFBP-2 protein levels within osteogenic cultures of ND and D BM-MSCs. Since the culture media was completely replaced on weekly bases, these higher concentrations reflect fresh weekly release by cultured BM-MSCs rather than accumulation. One plausible scenario is the diminished proteolytic breakdown of IGFBP-2 through the action of pregnancy-associated plasma protein A (PAPP-A), which was detected in cultures of DPSCs [12]. While PAPP-A is best known for targeting IGFBP-4, IGFBP-2 was also suggested as a potential substrate of PAPP-A [52,53]. Hence, future studies should investigate the gene and protein expression of PAPP-A and other matrix metalloproteinases to elucidate the effects of T2DM on the accumulation and bioavailability of the various components of the IGF axis. The lack of these analyses represents a limitation of the present study. Despite *IGFBP-3* downregulation in osteogenic vs. basal cultures, protein levels did not differ from basal conditions. This discrepancy could be attributed to the inhibition of IGFBP-3 proteinases, such as PAPP-A2 [54], producing these relatively higher concentrations of IGFBP-2 and -3. Like PAPP-A, PAPP-A2 is a metalloproteinase that selectively cleaves IGFBP-3 and -5, which are the major IGFBPs sequestering the IGFs in the circulation. As a result, PAPP-A2 can largely determine serum levels of the IGFs [54]. Likewise, the inhibition of IGFBP-4 proteases, particularly PAPP-A, either in long-term cultures or under osteogenic conditions, could potentially rationalise the markedly elevated IGFBP-4 levels at Wk2 vs. Wk1 basal cultures of ND BM-MSCs, and at Wk3 vs. Wk2 and Wk3 vs. Wk1 osteogenic cultures of ND BM-MSCs. While the major physiologic inhibitor of PAPP-A during pregnancy is the proform of eosinophil major basic protein (proMBP), stanniocalcin-2 (STC-2) is considered the major PAPP-A inhibitor in a non-pregnancy context [55]. There are other IGFBPs proteases such as matrix metalloproteinases (MMPs), which can target IGFBP-3 and -5; and cathepsins, which could equally cleave intracellular and extracellular IGFBPs [56]. By contrast, cleavage of IGFBP-4 seems to be highly specific to PAPP-A and rigidly dependent upon IGFBP-4 complexing with IGF-1 or -2 [57]. Furthermore, multiple pro-inflammatory cytokines, such as IL-1β and TNF-α, could promote PAPP-A expression as documented in multiple tissue injury models [57]. Whether this could ultimately affect the bioavailability of IGFBPs remains to be fully explored.

Our data showed that IGFBP-4 levels were elevated in osteogenic cultures of D BM-MSCs at Wk1 and ND BM-MSCs at Wk3 relative to their basal counterparts, possibly caused by PAPP-A downregulation or inhibition in osteogenic cultures. Conversely, Al-Khafaji et al. [12] observed *PAPP-A* upregulation and *STC-2* downregulation in osteogenic cultures of DPSCs. These seemingly contrasting findings could be attributable to the different cell origin (BM-MSCs in the present study vs. DPSCs). Moreover, Al-Khafaji et al. [12] cultured DPSCs for 1 week, a time point which demonstrated minimal changes in IGFBP concentrations in the present study. Nonetheless, future studies could aim at the quantification of transcript and protein levels of PAPP-A, -A2, and STC-2. This would provide deeper insights about the regulatory mechanisms governing IGFBP expression profiles in BM-MSC cultures in both healthy and diabetic conditions.

## 5. Conclusions

Although this in vitro study was limited by the relatively small sample size and the donors’ age and osteoarthritic status, it has shown that D BM-MSCs expressed lower levels of *IGFBP-2*, *-3* and *-4*. However, the protein release profiles of these IGFBPs in both D and ND cells were comparable. Modulating these molecules could prove promising for bone and periodontal regeneration using D BM-MSCs. Further research into possible roles of IGFBPs proteinases and their inhibitors during the osteogenic differentiation of BM-MSCs is warranted. Moreover, both D and ND BM-MSCs showed a trend of *IGFBP-2* upregulation in their osteogenic cultures along with a trend of *IGF-1* and *IGFBP-5* downregulation, and these molecules could represent potential targets to augment BM-MSC-based tissue regeneration.

## Figures and Tables

**Table 1:** Factors significantly impacting expression of IGF axis genes as outlined by 3-way ANOVA analysis.

IGF Axis Genes	Factors with Statistically Significant Impact (*p* < 0.05)
*IGF-1*	Diabetic status, culture media
*IGF-2*	--
*IGF1-R*	Diabetic status
*IGF2-R*	--
*IGFBP-1*	Diabetic status
*IGFBP-2*	Culture media
*IGFBP-3*	Diabetic status, culture media
*IGFBP-4*	Culture duration
*IGFBP-5*	Culture media
*IGFBP-6*	--

## Data Availability

Data will be made available upon request except for data that may compromise patients’ anonymity.

## Supplementary Materials

The following supporting information can be downloaded at https://www.mdpi.com/article/10.3390/bioengineering13091043/s1, Table S1: List of TaqMan^®^ gene expression assays used in qPCR.
