# Supplementary material for: Unravelling the Impact of Diabetes on Insulin-like Growth Factor (IGF) Axis Signalling in Mesenchymal Stem Cells Isolated from the Bone of Knee Joints
Source: Bioengineering (Basel). 2026 Sep 8;13(9):1043. doi: 10.3390/bioengineering13091043 (PMC13603204; doi:10.3390/bioengineering13091043)
Supplement: Supplementary file 1 [file bioengineering-13-01043-s001.zip › bioengineering-4310546-supplementary.pdf]

Table S1: List of TaqMan® gene expression assays used in qPCR

#	Gene	Description	TaqMan® Gene Expression Assay number
1	Hypoxanthine-guanine phosphoribosyl transferase 1 (*HPRT1*)	Housekeeping gene	Hs99999909_m1
2	Insulin like growth factor-1 (IGF-1)	Natural GF	Hs01547656_m1
3	Insulin like growth factor-2 (IGF-2)	Natural GF	Hs04188276_m1
4	Insulin like growth factor -1 receptor (IGF1-R)	Natural GF receptor	Hs00609566_m1
5	Insulin like growth factor-2 receptor (IGF2-R)	Natural GF receptor	Hs00974474_m1
6	Insulin like growth factor binding protein 1 (IGFBP-1)	Carrier protein for IGFs	Hs00236877_m1
7	Insulin like growth factor binding protein 2 (IGFBP-2)	Carrier protein for IGFs	Hs01040719_m1
8	Insulin like growth factor binding protein 3 (IGFBP-3)	Carrier protein for IGFs	Hs00426289_m1
9	Insulin like growth factor binding protein 4 (IGFBP-4)	Carrier protein for IGFs	Hs01057900_m1
10	Insulin like growth factor binding protein 5 (IGFBP-5)	Carrier protein for IGFs	Hs00181213_m1
11	Insulin like growth factor binding protein 6 (IGFBP-6)	Carrier protein for IGFs	Hs00181853_m1
